# Clinical outcomes of minimally invasive duodenum-preserving pancreatic head resection

**DOI:** 10.1186/s12893-023-02170-9

**Published:** 2023-09-21

**Authors:** Guangchen Zu, Weibo Chen, Di Wu, Yue Zhang, Xuemin Chen

**Affiliations:** Department of Hepatopancreatobiliary Surgery, The Third Affiliated Hospital of Soochow University, Changzhou, Jiangsu P.R. China

**Keywords:** Duodenum Preservation, Robotic surgery, Laparoscopy, Pancreatic head tumor

## Abstract

**Background:**

The procedure of total duodenum-preserving pancreatic head resection (DPPHRt) has been reported frequently, but rare in minimally invasive procedure, especially robotic-assisted operation. Here we share our experience and analyze the clinical outcomes of minimally invasive DPPHRt in the treatment of benign lesions or low-grade malignant tumors of the pancreatic head in this study.

**Materials and methods:**

From October 2016 to January 2022, three patients received robot-assisted DPPHRt(RA-DPPHRt), and seventeen patients received laparoscopic DPPHRt(LDPPHRt). Data were retrospectively collected in terms of demographic characteristics (age, gender, body mass index, and pathological diagnosis), intraoperative variables (operative time, estimated blood loss), and post-operative variables (post-operative hospital stay, and complications).

**Results:**

All 20 patients received minimally invasive total duodenum-preserving pancreatic head resection successfully without conversion, including 8 males and 12 females. Pathological diagnosis suggested 1 case of serous cystadenoma (SCA), 4 cases of intraductal papillary mucinous neoplasm (IPMN) ,5 cases of mucinous cystic neoplasm (MCN), 4 cases of pancreatic neuroendocrine neoplasm (PNET), 2 cases of chronic pancreatitis (CP),4 case of solid pseudopapillary tumor (SPT). The average operation time was (285.35 ± 95.13 min), ranging from 95 to 420 min. The average estimate blood loss was (196.50 ± 174.45ml) ,ranging from 10 to 600ml.The average post-operative hospital stay was(20.90 ± 14.44days),ranging from 8 to 54 days. Postoperative complications occurred in 10 patients (50%). A total of 5 patients (20%) suffered grade B or C pancreatic fistula. Two patients (10%) suffered from biliary fistula. Two patients (10%) suffered from delayed gastric emptying. One patient (5%) suffered from abdominal bleeding. The 90-day mortality was 0. No patient was observed tumor recurrence and new-onset diabetes but one developed diarrhea.

**Conclusion:**

RA-DPPHRt or LDPPHRt provided a minimally invasive approach with good organ-preservation for patients with benign and low-grade malignant pancreatic head tumor. It is only recommended to be performed in high-volume pancreatic centers by experienced pancreatic surgeons.

Duodenum-preserving pancreatic head resection (DPPHR) was first proposed by the German scholar Hans G Beger [1] to relieve the pain in patients with chronic pancreatitis. Compared to the traditional Whipple procedure, DPPHR successfully removed the lesions of the pancreatic head, improved the symptoms of pancreatitis, and preserved the continuity of the digestive tract to the greatest extent, avoiding cholangiojejunostomy, preserving the endocrine and exocrine functions of the pancreas [2]. Benefiting from the rapid progress in surgical instruments and surgical technique, minimally invasive pancreaticoduodenectomy (PD) and function-preserving pancreatic surgery have been reported in numerous medical centers. On the basis of carrying out great amount laparoscopic pancreaticoduodenectomy (LPD) previous [3], we completed minimally invasive DPPHRt for 20 patients successfully, using Da Vinci Xi or 3D laparoscopic equipment.

## Patients and methods

From October 2016 to January 2022,20 patients with benign or low-grade malignant pancreatic-head lesions received minimally invasive DPPHRt procedure in the Department of Hepatopancreatobiliary Surgery at the Third Affiliated Hospital of Soochow University. Three of them received robot-assisted DPPHRt(RA-DPPHRt), and the other 17 patients received laparoscopic DPPHRt(LDPPHRt).

The information, including the demographic data, preoperative examination results, pathological diagnosis, and short-term outcomes, such as operative time, estimated blood loss, postoperative hospitalization time, and complications such as postoperative pancreatic fistula (POPF), bile leakage, delayed gastric emptying (DGE), abdominal infection, and hemorrhage, were recorded and analyzed. The diagnosis of POPF and DGE was based on the criteria established by the International Study Group of Pancreatic Surgery (ISGPS) [4, 5].

All patients received abdominal enhanced CT, MR and endoscopic ultrasonography before surgery to clarify the type of lesion, the size of the lesion and the distance between the lesion boundary and the common bile duct (CBD) of the pancreatic segment. The present study was approved by the Ethics Committee of the Third Affiliated Hospital of Soochow University, and was performed in accordance with the relevant guidelines and regulations. Informed consent was obtained from all participants. SPSS 25.0 statistical software (IBM Corp, Armonk, NY, USA) was used for statistical analysis, and the data were expressed as mean ± standard deviation ($\overline x$±sd).

### Operative procedure

**Fig. 1:**
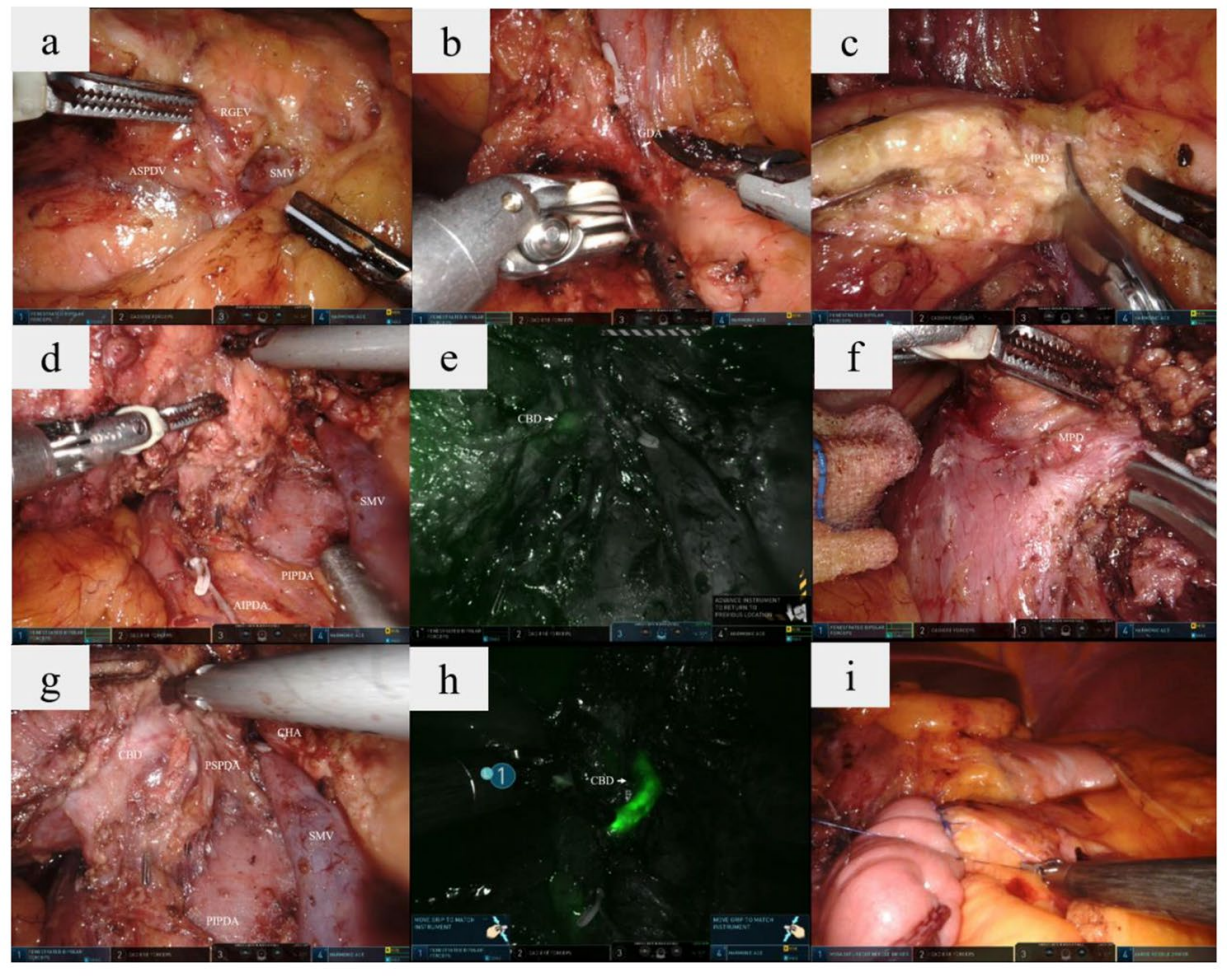
The specific surgical procedures involved in DPPHR. **(a)** RGEV and ASPDV inflow into SMV. **(b)** GDA was found on the upper border of the pancreas. **(c)** The main pancreatic duct was located and sheared. **(d)** The AIPDA and PIPDA were revealed. **(e)** The CBD was found with indocyanine green fluorescence imaging. **(f)** The main pancreatic duct in the ampulla of Vater was found, severed. **(g)** The PSPDA was exposed. **(h)** The CBD displayed in indocyanine green fluorescence imaging mode after pancreatic head resection. **(i)** The duct-to-mucosa pancreaticojejunostomy was carried out RGEV: right gastroepiploic vein; ASPDV: anterior superior pancreaticoduodenal vein; SMV: superior mesenteric vein; GDA: gastroduodenal artery; MPD: main pancreatic duct; CHA: common hepatic artery CBD: common bile duct; PIPDA: posterior inferior pancreaticoduodenal artery AIPDA: anterior inferior pancreaticoduodenal artery; PSPDA: posterior superior pancreaticoduodenal artery

**Fig. 2:**
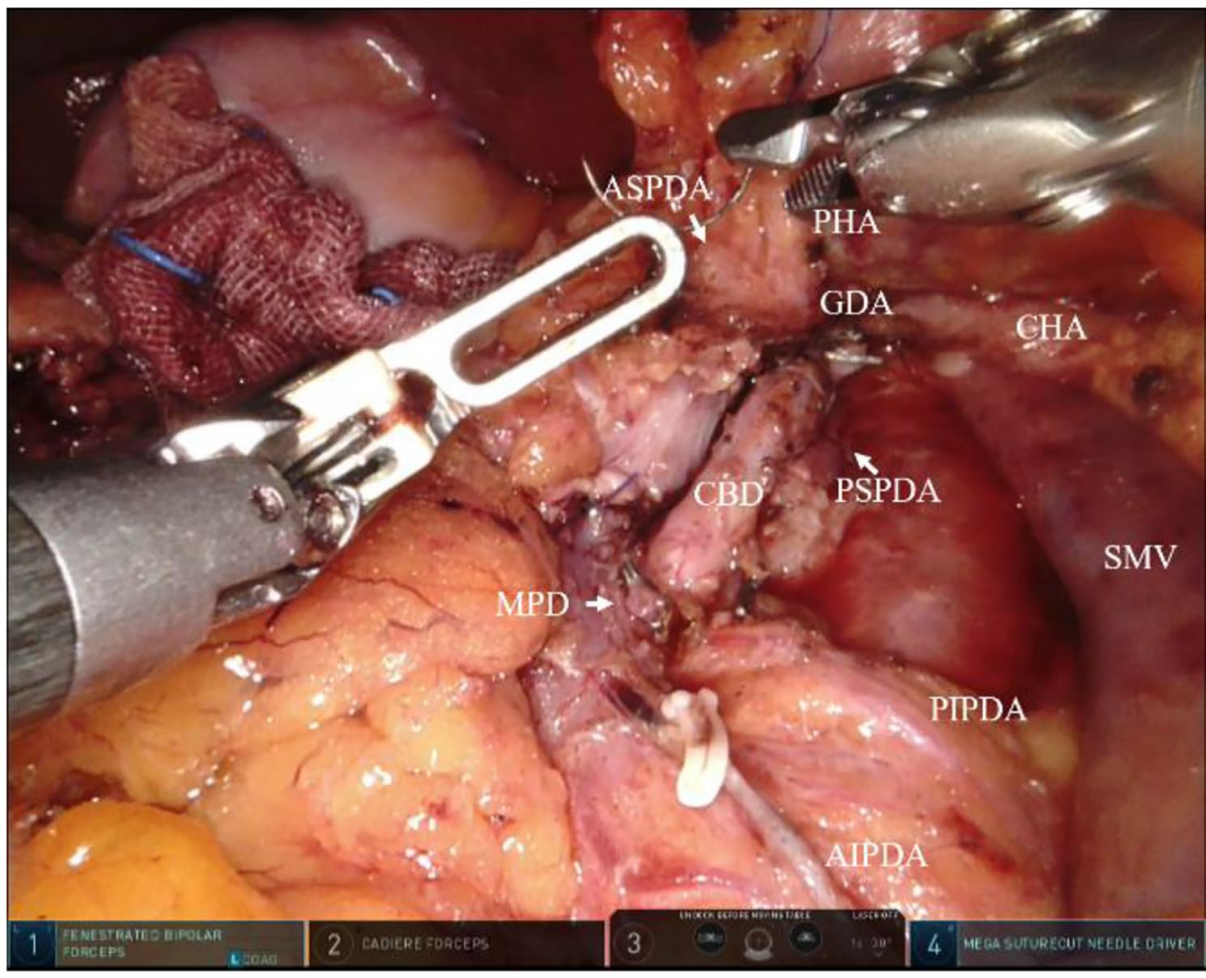
Operation area display after removing the pancreatic head ASPDA: anterior superior pancreaticoduodenal artery; PSPDA: posterior superior pancreaticoduodenal artery. PHA: proper hepatic artery; SMV: superior mesenteric vein; GDA: gastroduodenal artery; MPD: main pancreatic duct; CHA: common hepatic artery CBD: common bile duct; PIPDA: posterior inferior pancreaticoduodenal artery AIPDA: anterior inferior pancreaticoduodenal artery; SMV: superior mesenteric vein;

The DPPHRt surgical procedures were done as previously described [6]. The specific surgical procedures were showed in Fig. 1. Briefly, the pancreatic head and neck were exposed after the gastrocolic ligament was opened. The anterior superior pancreaticoduodenal vein (ASPDV) and the right gastroepiploic vein (RGEV) were then dissected (Fig. 1a), and the common hepatic artery (CHA), gastroduodenal artery (GDA), and superior mesenteric vein (SMV) were dissected from the superior and inferior margins of the pancreas (Fig. 1b). The post-pancreatic neck tunnel was created, and the pancreatic neck was transected along the right side of the portal vein (PV) using an ultrasonic scalpel. The main pancreatic duct was located and sheared (Fig. 1c), and the pancreatic head was pulled to the right to expose the right-side tract of the superior mesenteric artery (SMA). Dissection between pancreatic head and duodenum was performed under the pancreatic capsule from beginning to end. At this point, attention must be paid to protect the anterior inferior pancreaticoduodenal artery (AIPDA) and the posterior inferior pancreaticoduodenal artery (PIPDA) (Fig. 1d). The CBD was gradually revealed under the guidance of indocyanine green fluorescence imaging (Fig. 1e and h), and the main pancreatic duct in the ampulla of Vater was found, severed, and closed with a 5-mm titanium clip (Fig. 1f). The pancreatic tissue around the CBD was then dissected from bottom to top, and the anterior superior pancreaticoduodenal artery (ASPDA) and posterior superior pancreaticoduodenal artery (PSPDA) were exposed (Figs. 1g and 2). The bile duct wall should not be over-exposed. (9) A Roux-en-Y duct-to-mucosa pancreaticojejunostomy was then carried out after removing the specimen (Fig. 1i). Operation area after removing the pancreatic head were showed in Fig. 2.

## Results

### Patient characteristics

The patients’ characteristics are shown in the Table 1a &1b. A total of 20 patients were included in this study, including 8 males and 12 females. Three patients received RA-DPPHRt and the other seventeen patients received LDPPHRt, without conversion. The average age of these patients was 49.15 ± 17.46 years. The average body mass index(BMI)was 23.45 ± 4.22 kg/m^2^. The reasons for the patient’s visit included tumor found by imaging examination or enlarged tumor size in 12 cases, abdominal pain in 7 cases and recurrent hypoglycemia in 1 case. Seven patients had a history of abdominal surgery, 4 had hypertension, 1 had diabetes, and 1 had long-term oral hormones due to erythema annulare. American Society of Anesthesiologists score: 3 cases of grade I, 15 cases of grade II, and 2 cases of grade III.

**Table 1a:** Main characteristics of the 20 patients

Variables	
No. of patients	20
Sex (M/F)	8/12
Mean age (years)	49.15 ± 17.46
BMI (kg/m^2^)	23.45 ± 4.22
Pathological diagnosis	
SCA	1(5%)
IPMN	4(20%)
MCN	5(25%)
P-NET	4(20%)
CP	2(10%)
SPT	4(20%)

### Operative outcomes

The operative and post-operative outcomes are shown in Table 2a&2b. The average operation time was 285.35 ± 95.13 min (range 95–420 min). The average estimate blood loss was 196.50 ± 174.45ml (range 50-600ml). The average post-operative hospital stay was20.90 ± 14.44days (range 8–54 days). Postoperative complications occurred in 10 patients (50%), one of defined serious complication (Clavien-Dindo complication grade ≥ grade III). Ten patients (50%) suffered from pancreatic fistula, including 5 cases (25%) of biochemical leakage, 4 cases (15%) of grade B pancreatic fistula, and 1 case (5%) of grade C pancreatic fistula. Two patients (10%) suffered from biliary fistula. Two patients (10%) suffered from DGE. One patient (5%) suffered from abdominal bleeding. The 90-day mortality was 0. The mean postoperative follow-up time was 28.26 months, ranging from 6 to 66 months, and no patient was observed tumor recurrence and new-onset diabetes but one developed diarrhea.

**Table 1b:** Additional characteristics of the 20 patients

Patient no.		Reasons for visit		BMI (kg/ m^2^)		Abnormal blood levels of CA-19-9(u/ml)			Tumor’s Diameter (mm)		Diameter of the pancreatic duct(mm)			Mural nodules		Thickened and enhanced cystic wall		Count of high risk factors		Pathological diagnosis
1		Tumor found		20.83		29.60			30*28		5.2			No		No		3		SCA
2		Tumor found		25.64		No			25*10		11.4			No		No		2		MD-IPMN
3		Abdominal pain		22.03		No			15*10		9.5			No		No		1		MD-IPMN
4		Tumor found		20.42		39.66			35*10		6.0			No		No		3		MD-IPMN
5		Tumor found		20.57		No			30*28		13.7			No		No		2		MD-IPMN
6		Enlarged Tumor		33.87		No			54*35		2.2			Yes		Yes		3		MCN
7		Abdominal pain		17.58		No			35*30		7.3			No		No		1		MCN
8		Enlarged tumor		26.17		No			36*28		2.1			No		No		1		MCN
9		Abdominal pain		17.09		92.71			35*25		9.6			No		Yes		4		MCN
10*		Tumor found		23.88		No			32*15		11.1			No		No		3		MCN
11*		Hypoglycemia		23.88		No			15*10		2.0			No		No		-		P-NET,G1
12		Tumor found		22.72		No			25*12		1.8			No		No		-		P-NET,G1
13		Tumor found		29.30		No			33*27		2.0			No		No		-		P-NET,G2
14		Tumor found		27.43		No			42*40		2.1			No		No		-		P-NET,G2
15		Abdominal pain		25.71		No			30*24		5.0			No		No		-		CP
16		Abdominal pain		18.37		No			30*28		9.2			No		No		-		CP
17		Abdominal pain		28.08		No			57*47		1.1			Yes		No		2		SPT
18		Abdominal pain		23.66		No			35*32		2.0			No		No		2		SPT
19*		Enlarged tumor		20.20		No			30*25		1.8			No		Yes		2		SPT
20		Tumor found		21.51		No			34*30		1.9			No		No		1		SPT

F: female; M: male; BMI: body mass index; SCA: serous cystadenoma; MD-IPMN: Main duct,intraductal papillary mucinous neoplasm; MCN: mucinous cystic neoplasm; P-NET: pancreatic neuroendocrine tumors; CP: chronic pancreatitis; SPT: solid pseudopapillary tumors; mm: millimeter;

high risk factors: lesion size greater than 3 cm, presence of mural nodules, dilation of the main pancreatic duct , excess weight (BMI≥23kg/m^2^ ,according to Asia-Pacific guidelines), abnormal blood levels of CA-19-9;

*: represent patients receive robotic surgery

**Table 2a:** Operative and post-operative outcomes of RA-DPPHRt

Patient no.	Operation time(min)	Blood loss(ml)	POPF	Bleeding	DGE	Pancreatitis	Biliary fistula	Clavien-Dindo grade ≥ 3	Conversion	Re-operation
10	420	100	C	Yes	No	No	Yes	Yes	No	No
11	324	120	BF	No	No	No	No	No	No	No
19	300	90	No	No	No	No	No	No	No	No

POPF: postoperative pancreatic fistula; BF: biochemical fistula; DGE: delayed gastric emptying

**Table 2b:** Operative and post-operative outcomes

Variables	n, %
Type of DPPHR	
RA-DPPHRt	3, 15%
LDPPHRt	17, 75%
Postoperative local complications	
POPF	10, 50%
Biochemical fistula	5, 25%
Grade B	3, 15%
Grade C	1, 10%
Bleeding	1, 10%
DGE	2, 10%
Pancreatitis	0, 0%
Biliary fistula	2, 10%
Clavien-Dindo grade ≥ 3	1, 5%
Conversion	0, 0%
Re-operation	0, 0%
90-day mortality	0, 0%

DPPHRt: total duodenum-preserving pancreatic head resection; RA-DPPHRt: robot-assisted DPPHRt; LDPPHRt: laparoscopic DPPHRt; POPF: postoperative pancreatic fistula; DGE: delayed gastric emptying

### Pathological diagnosis

The pathological diagnosis are shown in the Table 1a and Table 1b.

Among the 20 cases in this study, there were 1 case (5%) of SCA, 4 cases (20%) of IPMN, 5 cases (25%) of MCN, 4 cases (20%) P-NET, 2 cases (10%) of chronic pancreatitis, 4 case (20%) of SPT.

## Discussion

The benign or low-grade malignant tumors of the pancreatic head were more frequently detected, with the increase of awareness and widespread use of high-resolution cross-sectional.

imaging [7]. In this study, pancreatic cystic tumors accounted for 70% (14/20), female patients accounted for 60% (12/20), and the mean age was 49.15 ± 17.46 years. These parameters suggest that middle-aged female patients were the predominant crowd in this study, and benign pancreatic cystic lesions were the main lesion type. There are 2 major challenges when managing pancreatic cysts. The first is to discriminate benign cysts from cysts with the potential to develop into pancreatic ductal adenocarcinoma (PDAC). The second is to determine the risk of progression to highgrade dysplasia (HGD) or invasive PDAC in patients with mucinous cysts (IPMNs and MCNs) [8, 9]. Therefore, surgeons should be very careful in making decisions for patients with cystic pancreatic lesions to avoid unnecessary surgeries and missed patients with potential pancreatic cancer. According to the global guidelines on pancreatic cystic lesions reported by World Gastroenterology Organization (WGO) [10], patients with at least two of these risk factors have about a 15% chance of developing pancreatic malignancy: lesion size greater than 3 cm, presence of mural nodules, dilation of the main pancreatic duct. Other factors may also be predictive of a higher risk of malignancy: family history of pancreatic cancer, mutations that predispose to pancreatic cancer, abnormal blood levels of CA-19-9, unexplained acute pancreatitis, especially in patients aged > 50 y, recent-onset diabetes mellitus, excess weight, low serum levels of pancreatic amylase and lipase, coarse calcification. Therefore, we followed the above principles to make individualized surgical strategy for patients who sufferred from pancreatic cystic neoplasms(PCNs).The pathological results suggested 1 case of SCA in this study, and this patient was diagnosed as P-NET in the preoperative evaluation, for being observed abundant blood flow signals in the tumor by enhanced abdominal CT and MR. This case reminds us to closely evaluate the patients’ diagnosis before surgery, and it is necessary to repeatedly read the imaging data at certain times to ensure a low rate of unnecessary operation.

As early-stage pancreatic cancer patients have mild or no clinical symptom, most pancreatic cancer patients are diagnosed at an advanced stage of cancer after obvious symptoms appear or during physical examination and hardly undergo radical resection [11]. In this study, 12 asymptomatic patients seek medical advice for tumor found by imaging examination or enlarged tumor size. Although they were asymptomatic, this does not mean that they were perfectly healthy and could be easily ignored. For patients with surgical indications, surgical treatment were recommended to avoid missed diagnosis of pancreatic cancer.

In contrast to malignant pancreatic tumors, these cystic tumors occur typically in young women, a less invasive and faster recovery surgical procedure is needed. DPPHR has turned out to be an optimal option for the treatment of these pancreatic diseases compared with PD [12]. DPPHR could preserves the continuity of the digestive tract to the greatest extent, retains the hormone secretion function of the digestive tract in the duodenum and proximal jejunum, and it has obviously advantages in maintaining the endocrine and exocrine functions of the pancreas, which can effectively reduce surgical trauma and improve postoperative recovery [13]. Minimally invasive surgery should be considered as a preferred treatment option for those patients with long-life expectancies and with possibility for a higher post-operative quality of life [14, 15].

However, the anatomy of this operation is too complex and difficult for a beginner to operate, especially in minimally invasive technology. LDPPHRt or RA-DPPHRt has been reported by several scholars in recent years, the results indicated that this minimally invasive procedure is feasible and safety [16–18]. The preservation of the blood supply to the bile duct and the duodenum is the hinge to the procedure. It is reported that the main blood supply consists of anterior and posterior pancreatic duodenal arterial arcades with a bidirectional blood flow [19]. Kim et al. supposed that the posterior pancreaticoduodenal artery provides the major blood supply to.

the papilla and distal bile duct and preserving the whole posterior pancreaticoduodenal artery and anterior inferior pancreaticoduodenal artery was recommended [20]. Cao J et al. presented by autopsy that the anterior pancreatic duodenal arterial arcade runs typically in the capsule of the pancreas and the posterior pancreatic duodenal arterial arcade runs in the mesopancreas [18].Therefore ,we took similar strategies to protect the arterial arcades, for instance, dissecting the pancreatic parenchyma inside the capsule to protect anterior pancreatic duodenal arterial arcade, and the Kocher’s maneuver was not recommended to perform for preserving the posterior arcade running in the mesopancreas [14, 18]. The whole posterior pancreaticoduodenal artery and AIPDA were preserved but the ASPDA would be resected if necessary.

Pancreatic fistula is the most common postoperative complication, Beger et at. through meta-analysis reported the incidence of POPF is 19.2–22.1% in various DPPHR, and the incidence of grade B/C pancreatic fistula is 10.7–13.6% [2, 21]. In contrast to Beger’s statistical results, several scholars reported their overall incidence of POPF is 32.4–45.8% but the incidence of grade B/C pancreatic fistula was 4.2–19.8% [14, 17, 22], similarly to our study outcome (50% and 20%, respectively). The reason caused such difference may be the different reading about the POPF, since the definition of POPF was revised by the ISGPS in 2017. In addition, we usually preserve a thin layer of pancreatic tissue around the CBD to protect its nourishing blood vessels, contributing to the relatively high incidence of POPF.

Compared to PD, biliary leak and bile duct stricture occurred more frequently in DPPHR [14, 23]. Beger et al. reviewed postoperative morbidity following DPPHR, found that DPPHR had a higher biliary complication rate than duodenal segment resection (DPPHR-S) (8.4% vs. 0.7%) [21]. In LDPPHR, bile leakage occurred in 2 of the 12 patients (16.7%) in a study by Cao et al. [18], and 3 of 24(12.5%) by Cai et al. [14]. Moreover, Jiang Y et al. reported that the incidence of bile leakage of RA-DPPHR was 11.8%(4/34). And our study outcomes showed the bile leakage occurred in 2 of the 20 patients (10%). The incidence of biliary leak was usually associated with thermal damage to the bile duct wall caused by the ultrasonic knife, and the destruction of the feeding vessels of CBD due to excessive denudation of the bile duct wall, as well as the failure to preserve posterior pancreaticoduodenal arcade [21]. To avoided bile duct injury, indocyanine green(ICG) -enhanced fluorescence imaging was used in this study. Under the ICG-enhanced fluorescence imaging, biliary and vascular structures could be clearly recognized, and the surgeon had a precise view of the anatomy [24]. Furthermore, most patients who suffered from biliary leak or bile duct stricture recovered via placement of a biliary stent under endoscopy; only a few needed further surgical procedures such as T-tube drainage or PD.

In this study, 1 patient suffered from biliary leak and pancreatic fistula (grade C) and abdominal bleeding meanwhile. The biliary leak and pancreatic fistula were significantly improved by percutaneous gallbladder drainage (PTGBD) and long-term preservation of abdominal drainage. The abdominal bleeding occurred on the days 15 and 28 after surgery, and terminated after a series of conservative treatment including plasma and red blood cell transfusion as well as application of hemostatic drugs, the patient finally recovered from the severe hemorrhagic shock without being performed emergent exploratory laparotomy. The BMI of this patient is 29.3 kg/m^2^, which is much higher than the others. In addition, this patient continuously took prednisone 5 mg/day to treat dermatomyositis for more than 20 years. Those special factors may cause anatomy more challenging and bring about secondary injury during the operation [25]. There were some limitations in this study. First, this is a single-center, small-sample retrospective study. The results and conclusions need to be further verified by a prospective randomized controlled trial with enough sample size. Second, we did not compare the robotic surgery group with the laparoscopic surgery group, due to the small number of cases in the robotic surgery group. Additionally, the advantages of minimally invasive surgery on long-term outcomes, such as CBD stenosis, cholestasis, and jaundice, to be confirmed by further research.

## Conclusion

RA-DPPHRt or LDPPHRt provided a minimally invasive approach with good organ-preservation for patients with benign and low-grade malignant pancreatic head tumor. Considering the complexity and high risks involved, it is only recommended to be performed in high-volume pancreatic centers by experienced pancreatic surgeons. The long-term outcomes of minimally invasive surgery and the quality of life of these patients require further study and discussion.

## Data Availability

The datasets used during the current study are available from the corresponding author on reasonable request.
